# Elevated Systemic and Intestinal Inflammatory Response Are Associated With Gut Microbiome Disorder After Cardiovascular Surgery

**DOI:** 10.3389/fmicb.2021.686648

**Published:** 2021-08-25

**Authors:** Xiong Xia, Jiangjin Ni, Shengnan Yin, Zhipeng Yang, Haini Jiang, Chao Wang, Jian Peng, Hongkui Wei, Xingyu Wang

**Affiliations:** ^1^Department of Animal Nutrition and Feed Science, College of Animal Science, Huazhong Agricultural University, Wuhan, China; ^2^Medical Affairs Office, Tongji Hospital, Tongji Medical College, Huazhong University of Science and Technology, Wuhan, China; ^3^Division of Cardiothoracic and Vascular Surgery, Tongji Hospital, Tongji Medical College, Huazhong University of Science and Technology, Wuhan, China

**Keywords:** cardiovascular diseases, perioperation, gut microbiome, systemic inflammatory response, gut barrier, gut local inflammation

## Abstract

Systemic inflammatory response after cardiovascular surgery is associated with poor prognosis, to which gut barrier impairment is related. To investigate whether perioperative changes of the gut microbiome are associated with systemic and intestinal inflammatory response, we examined changes of the gut microbiome, intestinal homeostasis, and systemic inflammatory response in cardiovascular patients before (Pre) surgery and on the first defecation day [postoperative time 1 (Po1)] or a week [postoperative time 2 (Po2)] postsurgery. Markedly, the enhanced systemic inflammatory response was observed in Po1 and Po2 compared with that in Pre. In line with inflammatory response, impaired gut barrier and elevated gut local inflammation were observed in Po1 and Po2. Microbiome analysis showed a remarkable and steady decline of alpha diversity perioperatively. In addition, microbial composition in the postoperation period was characterized by significant expansion of *Enterococcus* along with a decrease in anaerobes (*Blautia*, *Faecalibacterium*, *Bifidobacterium*, *Roseburia*, *Gemmiger*, [*Ruminococcus*], and *Coprococcus*), which were typically health-associated bacteria. Spearman correlation analysis showed microbiome disorder was associated with enhanced systemic inflammatory response and intestinal dysbiosis. These results suggest that microbiome disorder was related to disturbed gut homeostatic and subsequently elevates plasma endotoxin and systemic inflammatory response after cardiovascular surgery. This study not only highlights gut microbiome would be considered in future clinical practice but also proposes a promising perspective of potential diagnostic and therapeutic options for perioperative management of cardiovascular surgery patients.

## Introduction

The incidence of cardiovascular disease is increasing in recent years (Roth et al., 2017). Patients with cardiovascular diseases who suffered from acute or chronic cardiac insufficiency induced by structural abnormality are usually in need of surgical treatment (Du et al., 2019). During cardiovascular surgery, multiple factors, such as cardiopulmonary bypass, anesthesia, ischemia–reperfusion injury, blood transfusion, etc., could precipitate systemic inflammatory response. The elevated systemic inflammatory response is commonly attributed to postoperative complications and poor prognoses, such as prolonged hospital stays and increased mortality (Kraft et al., 2015).

Previous studies had observed impaired gut barrier among patients after cardiovascular surgery, and systematic inflammation was one of the major reasons (Lau et al., 2000; Morty et al., 2008; Kano et al., 2017). It has been gradually recognized that pro-inflammatory cytokines can lead to disturbance in the intestinal barrier, causing increased tissue penetration of luminal antigens and the development of intestinal and systemic inflammation (Al-Sadi et al., 2013). Thus, bacterial endotoxin from the gut might be one contributor to inflammation (Gorla et al., 2018). However, further study regarding microbiome change during the perioperative period is infrequent.

Collaboration between the host and the human gut microbiome plays key roles in health (Fassarella et al., 2020), whereas gut microbiome dysbiosis, such as increased *Proteobacteria* and decreased *Firmicutes*, would lead to intestinal inflammation, resulting in a permeable gut and causing microbial-related molecules penetration and translocation (Wei et al., 2016; Mouries et al., 2019; Zhu et al., 2019). Also, this process would trigger systemic inflammation and is subject to the development of various gastrointestinal diseases (Wei et al., 2016; Fassarella et al., 2020). However, rare studies have revealed the link between gut microbiome with gut homeostatic and systemic inflammatory response during the perioperative period of cardiovascular surgery.

The current study was conducted to explore the perioperative change of systematic inflammatory response, gut permeability, intestinal inflammation and immunity, and gut microbiome of patients undergoing cardiovascular surgery. This study aimed to elucidate potential relative among gut inflammation, microbiome, and systemic inflammatory response and provide a foundation for perioperative management of cardiovascular surgery patients based on gut microbiota. We hypothesize that enhanced systemic inflammatory response and gut dysbiosis are associated with gut microbiome disorder after cardiovascular surgery.

## Materials and Methods

### Ethics and Patients

This study was approved by the Ethics Committee of Tongji Hospital (TJ-IRB20210203), Tongji Medical College of Huazhong University of Science and Technology (Wuhan, Hubei, China) and conducted in accordance with the Declaration of Helsinki. Informed consent was obtained from the participants and/or their families.

A cohort of 67 patients receiving cardiovascular surgery was consecutively recruited for this study from Tongji Hospital. Their serum and/or stool samples were collected. The preoperative diagnosis included congenital heart disease, coronary heart disease, end-stage heart diseases, heart valvular disease, and aortic dissection. Inclusion criteria were as follows: (1) age >18 years and ≤80 years, (2) patients diagnosed with a cardiovascular disease requiring surgical treatment, (3) voluntarily signed informed consent, and (4) no antibiotic and prebiotic intake within the last month preceded to admission. Exclusion criteria were as follows: (1) participated in other clinical trials within the last 3 months, (2) pregnancy or breastfeeding women, (3) gastrointestinal diseases that affect the gut microbiome such as inflammatory bowel disease, irritable bowel syndrome, celiac disease, and/or so on, and (4) diarrhea history within 1 month before admission.

### Data Collection

Perioperative laboratory values and clinical information were collected from medical records. Preoperative data, which consisted of sex, age at operation, body weight, height, body mass index, hypertension, diabetes mellitus, history of smoking and/or alcohol, peripheral arterial disease, left ventricular ejection fraction (LVEF), hyperlipidemia, chronic renal insufficiency, anemia, atrial fibrillation, and pulmonary hypertension, were obtained. White blood cell (WBC) and the ratio of neutrophil count (Neu%), high-sensitivity C-reaction protein (hs-CRP), and procalcitonin (PCT) were obtained before surgery (Pre), first defecation day [postoperative time 1 (Po1)], and a week [postoperative time 2 (Po2)] after surgery. Additionally, unexpected reoperation for bleeding, length of hospital stays or intensive care unit (ICU) stay, and inpatient mortality were deemed major clinical outcomes in this study.

### Sample Processing

Paired peripheral blood and fecal samples were collected from 67 recruited patients at Pre, Po1, and Po2, and a total of 181 paired blood and fecal samples were collected. Specifically, the sample size of blood and fecal samples for Pre, Po1, and Po2 were 55, 66, and 60, respectively. The blood samples were immediately stored on ice after being collected, and plasma was isolated within 30 min by centrifugation at 3000 × *g* for 10 min at 4°C. Plasma was immediately stored at −80°C in aliquots until analysis. Each inpatient was provided with a sterile stool specimen collection box during hospitalization and asked to collect stools at the three periods mentioned previously. Samples were stored at −80°C for further analysis.

### Biochemical Analysis

Plasma level of tumor necrosis factor-α (TNF-α), interleukin-6 (IL-6), and zonulin and fecal level of β-defensins 2 were determined by using human ELISA kits (ABclonal, Wuhan, China). Plasma level of lipopolysaccharides (LPS), soluble CD14 (sCD14), and intestinal fatty binding acid (iFABP) and fecal level of lipocalin-2, calprotectin, and sIgA were measured by using human ELISA kits (Mlbio good ELISA kit producers, Shanghai, China). All measurements were performed according to the manufacturer’s instructions.

### DNA Extraction, 16S Ribosomal RNA Gene Amplification, and Illumina MiSeq Sequencing

Microbial DNA was extracted from intestinal contents using a QIAamp DNA Stool Mini Kit (Qiagen, Germany) following the manufacturer’s protocols. Successful DNA extraction was confirmed by 0.8% agarose gel electrophoresis. The V3–V4 hypervariable region of the bacterial 16S ribosomal RNA (rRNA) gene was amplified using primers 341F (5′-ACT CCT ACG GGA GGC AGC AG-3′) and 806R (5′-GGA CTACHV GGG TWT CTA AT-3′). The polymerase chain reaction (PCR) conditions were predenaturation at 98°C for 2 min, 25 cycles of denaturation at 98°C for 15 s, annealing at 55°C for 30 s, elongation at 72°C for 30 s, and a final post-elongation cycle at 72°C for 5 min. The PCR products were purified with AMPure XP beads (Axygen). After purification, the PCR products were used for the construction of libraries and then paired-end sequenced on Illumina MiSeq (Illumina, CA, United States) at the Personalbio, Shanghai, China.

### Sequence Filtering, Amplicon Sequence Variant Clustering, and Sequence Analyses

Microbiome bioinformatics was performed with QIIME 2 2019.4 (Bolyen et al., 2019) with slight modification according to the official tutorials. Briefly, raw sequence data were demultiplexed using the demux plugin following by primers cutting with the cutadapt plugin (Martin, 2011). Sequences were then quality filtered, denoised, merged, and chimera removed using the DADA2 plugin (Callahan et al., 2016). Non-singleton amplicon sequence variants were aligned with mafft (Katoh et al., 2002) and used to construct a phylogeny with fastTree2 (Price et al., 2010). Taxonomy was assigned to amplicon sequence variants using the classify-sklearn naïve Bayes taxonomy classifier in the feature-classifier plugin (Bokulich et al., 2018) against the Greengenes 13_8 99% operational taxonomic unit reference sequences (McDonald et al., 2012). Alpha diversity values of each sample were assessed on the Chao richness estimator (Chao1) and Shannon index. Beta diversity measures depended on unweighted UniFrac distance. Linear discriminant analysis coupled with effect size was conducted to identify bacterial taxa differentially represented between different stages at genus or higher taxonomy levels (Segata et al., 2011). Organism level microbiome phenotypes were predicted and compared with BugBase (Langille et al., 2013).

### Statistical Analyses

Before analysis, Shapiro–Wilk and Levene tests were performed for the normality and heteroscedasticity of continuous data (with the significance level set at 5%). The difference among the three groups was performed using unpaired one-way analysis of variance or Kruskal–Wallis test for a parametric or non-parametric test, followed by a *post hoc* test using Dunn’s multiple comparison test. Correlations were analyzed using Spearman’s correlation in GraphPad Prism (GraphPad Software, San Diego, CA, United States). In the figures, *P* < 0.05 indicates statistical significance (^∗^*P* < 0.05, ^∗∗^*P* < 0.01, and ^∗∗∗^*P* < 0.001). Analyses were performed using R (R Core Team, Vienna, Austria), GraphPad Prism (version 8.0.1, GraphPad Software Inc., La Jolla, CA, United States), and SAS (version 9.4; SAS Institute Inc., Cary, NC, United States).

## Results

### Characteristics of the Study Population

The demographic data of the study cohort are shown in Table 1. The cohort had a median age of 55 years, and 41 (61.19%) patients were male. Peripheral arterial disease (40.3%), anemia (22.39%), and hypertension (17.91%) were the leading preoperative concomitants of the cohort. For all the patients, median preoperative LVEF was 65%. The hospital stay and median ICU stay of the study cohort were 14 days and 69.1 h. Two patients suffered from unexpected reoperation for bleeding. The inhospital mortality was 4.48%.

**TABLE 1:** Demographic data of study population.

Item			Total (*n* = 67)
Preoperative status			
		Male sex (*n*, %)	41 (61.19)
		Age (years)	55 (45.5–63.0)
		Height (cm)	168 (161.5–173.0)
		Weight (kg)	63 (58–72)
		BMI (kg/m^2^)	22.7 (21.4–25.1)
		Smoking history (*n*, %)	8 (11.94)
		Alcohol history (*n*, %)	7 (10.45)
		Diabetes mellitus (*n*, %)	9 (13.43)
		Hypertension (*n*, %)	12 (17.91)
		Hyperlipidemia (*n*, %)	6 (8.96)
		Anemia (*n*, %)	15 (22.39)
		Chronic renal insufficiency (*n*, %)	7 (10.45)
		Peripheral arterial disease (*n*, %)	27 (40.3)
		Atrial fibrillation (*n*, %)	5 (7.46)
		Pulmonary hypertension (*n*, %)	6 (8.96)
		LVEF (%)	65 (58.5–71.0)
Clinical outcomes			
		Hospital stay (days)	14 (12–22)
		ICU stay (hours)	69.1 (36.2–115.1)
		Unexpected reoperation for bleeding (*n*, %)	2 (2.99)
		In-patient mortality (*n*, %)	3 (4.48)

*BMI, body mass index; LVEF, left ventricular ejection fraction; ICU, intensive care unit.*

### Enhanced Systemic Inflammatory Response After Cardiovascular Surgery

**FIGURE 1:**
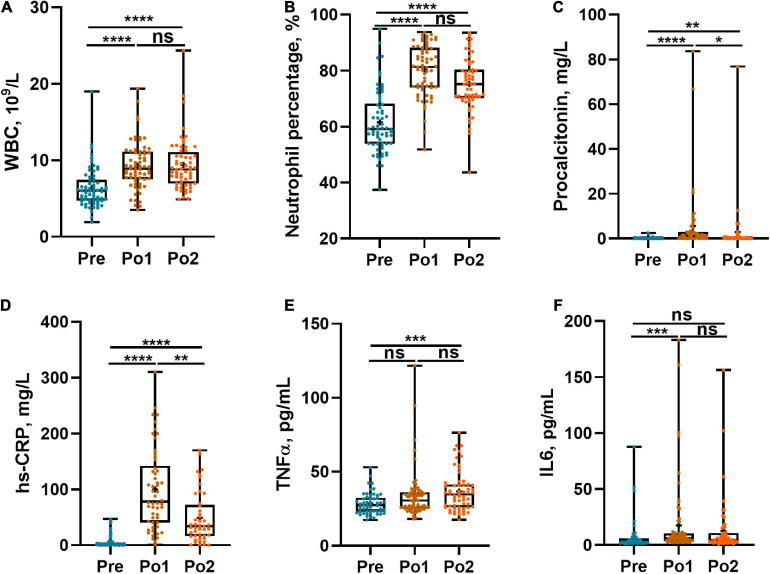
Perioperative systemic infection biomarkers of cohorts. WBC **(A)**, percentage of neutrophil count **(B)**, PCT **(C)**, hs-CRP **(D)**, TNF-α **(E)**, and IL-6 **(F)** were assessed across time series. Statistical assessment was carried out with one-way analysis of variance or Kruskal–Wallis test for parametric or non-parametric data followed by *post hoc* test using Dunn’s multiple comparison test. *P* < 0.05 indicates statistical significance (*P < 0.05, **P < 0.01, ***P < 0.001, and ****P < 0.0001), and ns means *P* > 0.05. Results are expressed as median and quartile.

To determine the change of inflammation status during the perioperation period, WBC count, Neu%, serum PCT and hs-CRP, and plasma TNF-α and IL-6 were assessed. WBC count and Neu% were remarkably higher in postoperation time whereas showing no difference between Po1 and Po2 (Figures 1A,B). Serum PCT and hs-CRP levels were obviously elevated in Po1 and then descended in Po2, whereas the level of Po2 was still higher than that in Pre (Figures 1C,D). Meanwhile, plasma TNF-α was higher in Po2 than in Pre (Figure 1E), and plasma IL-6 was higher in Po1 than Pre (Figure 1F). These results indicated that systemic inflammation response was profoundly enhanced after surgery.

### Elevated Intestinal Inflammation and Disturbed Gut Immunity After Cardiovascular Surgery

**FIGURE 2:**
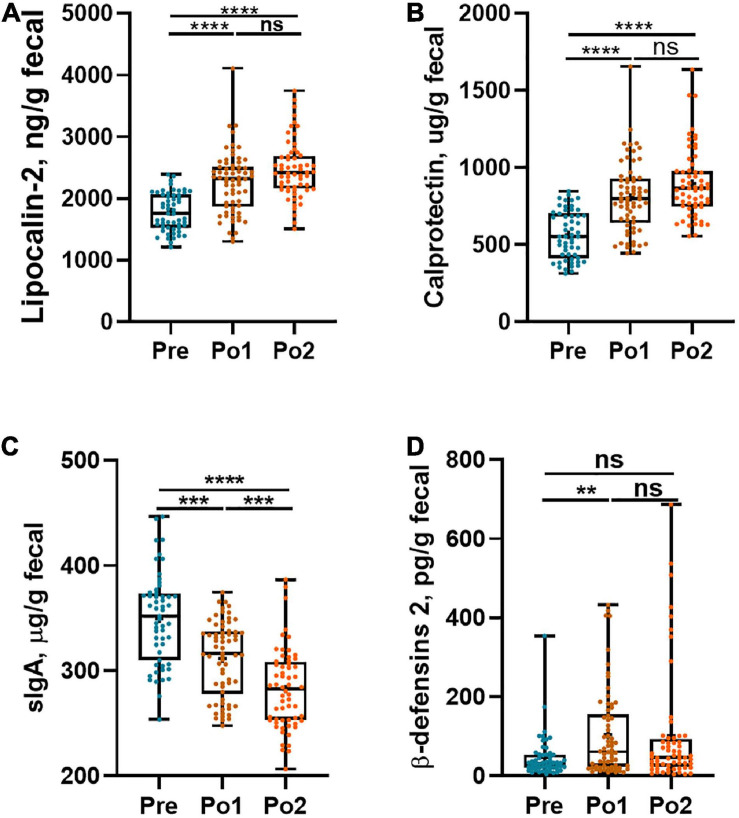
Perioperative intestinal local inflammation and immunity biomarkers of cohorts. Fecal lipocalin-2 **(A)**, calprotectin **(B)**, sIgA **(C)**, and β-defensins-2 **(D)** were assessed across time series. Statistical assessment was carried out with one-way analysis of variance or Kruskal–Wallis test for parametric or non-parametric data followed by *post hoc* test using Dunn’s multiple comparison test. *P* < 0.05 indicates statistical significance (*P < 0.05, **P < 0.01, ***P < 0.001, and ****P < 0.0001), and ns means *P* > 0.05. Results are expressed as median and quartile.

Perioperative intestinal inflammation and gut immunity were evaluated by fecal lipocalin-2, calprotectin, β-defensins 2, and sIgA, which are shown in Figure 2. Lipocalin-2 and calprotectin levels in Po1 and Po2 were profoundly higher than those in Pre and showed no difference between Po1 and Po2 (Figures 2A,B), whereas fecal sIgA steadily decreased in the same period (Figure 2C). However, β-defensins 2 in Po1 was higher than in Pre, but the level of Po2 showed no difference from Pre and Po1 (Figure 2D). Taken together, local gut inflammation was enhanced, and gut immunity was disturbed postsurgery.

### Impaired Gut Barrier After Cardiovascular Surgery

**FIGURE 3:**
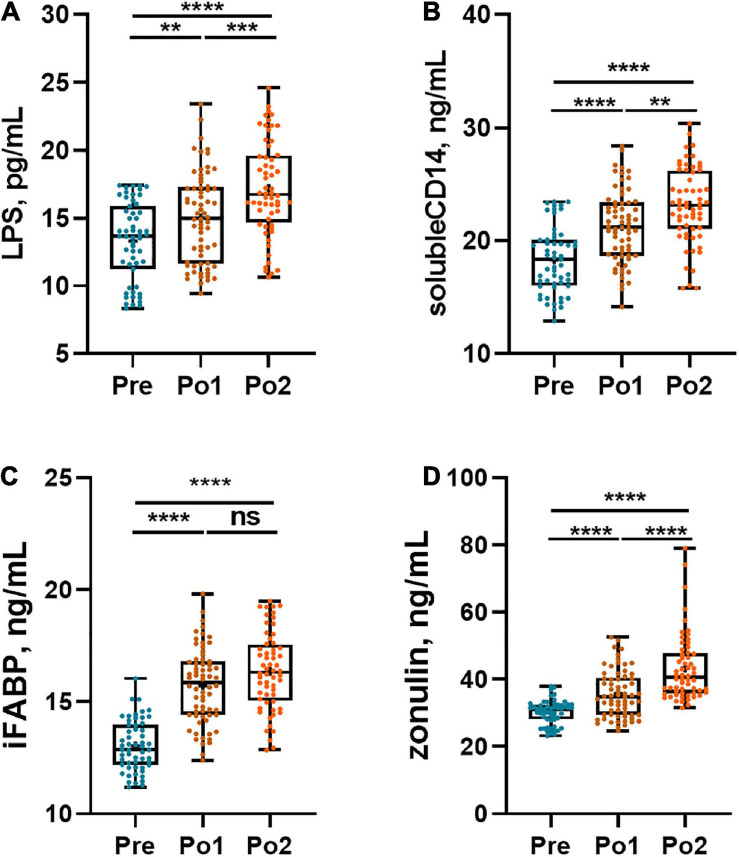
Perioperative gut barrier biomarkers of cohorts. Plasma LPS **(A)**, sCD14 **(B)**, iFABP **(C)**, and zonulin **(D)** were assessed across time series. Statistical assessment was carried out with one-way analysis of variance or Kruskal–Wallis test for parametric or non-parametric data followed by *post hoc* test using Dunn’s multiple comparison test. *P* < 0.05 indicates statistical significance (*P < 0.05, **P < 0.01, ***P < 0.001, and ****P < 0.0001), and ns means *P* > 0.05. Results are expressed as median and quartile.

The gut barrier was evaluated by plasma LPS, sCD14, iFABP, and zonulin, and the results are shown in Figure 3. Plasma LPS and sCD14 were continuously increased postoperatively (Figures 3A,B). Furthermore, the iFABP levels in Po1 and Po2 were strikingly higher than those in Pre, and no difference was observed between Po1 and Po2 (Figure 3C). Likewise, the plasma level of zonulin was also gradually increased during perioperation (Figure 3D). Taken together, patients showed postoperatively aggravated intestinal permeability and impaired intestinal epithelial integrity.

### Disordered Fecal Microbiome After Cardiovascular Surgery

**FIGURE 4:**
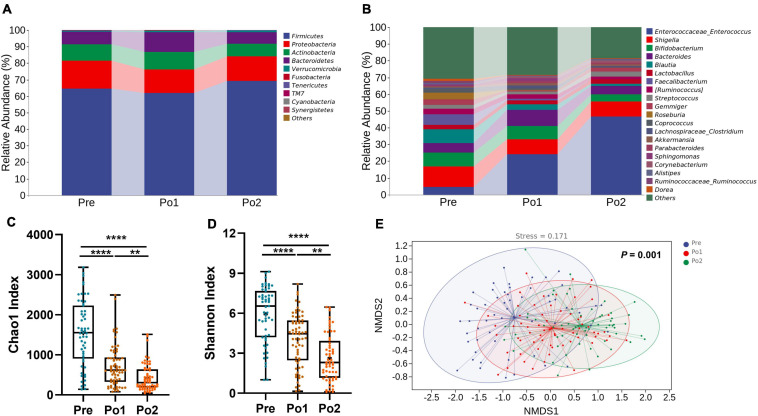
Perioperative change of microbial taxonomy, alpha diversity, and beta diversity. Phylum **(A)**, genus **(B)** Chao1 index **(C)**, Shannon index **(D)**, and non-metric multidimensional scaling analysis **(E)** were assessed across time series. Statistical assessment of Chao1 and Shannon index was carried out with Kruskal–Wallis test for parametric or non-parametric data followed by *post hoc* test using Dunn’s multiple comparison test. Analysis of similarities (ANOSIM) was performed to test statistical differences. *P* < 0.05 indicates statistical significance (*P < 0.05, **P < 0.01, ***P < 0.001, and ****P < 0.0001), and ns means *P* > 0.05. Results are expressed as median and quartile.

All fecal samples were subjected to 16S rRNA gene sequencing. The top 10 phyla and top 20 genera in the relative abundance of fecal microbiome presented in patients are displayed in Figures 4A,B. *Firmicutes* (65.20%) and *Proteobacteria* (15.48%) were the most dominated phyla in patients, followed by *Actinobacteria* (9.29%), *Bacteroidetes* (8.74%), *Verrucomicrobia* (0.93%), and *Fusobacteria* (0.13%). At the genus level, *Enterococcus* (25.08%), *Shigella* (10.09%), *Bifidobacterium* (6.87%), *Bacteroides* (6.75%), *Blautia* (4.29%), *Lactobacillus* (2.51%), *Faecalibacterium* (2.48%), [*Ruminococcus*] (2.47%), *Streptococcus* (2.29%), and *Gemmiger* (2.19%) were the 10 most abundant genera.

We then assessed the alpha and beta diversity of patients during the perioperation period. The Chao1 index and Shannon index were persistently dropping perioperatively (Figures 4C,D). Through non-metric multidimensional scaling based on unweighted UniFrac distance and tested by analysis of similarities (ANOSIM), we found that the gut microbiome of patients showed significant and orderly segregation (Figure 4E). Taken all together, the fecal microbiome of patients was significantly disordered during perioperation.

### Disturbed Phenotype of Fecal Microbiome After Cardiovascular Surgery

**FIGURE 5:**
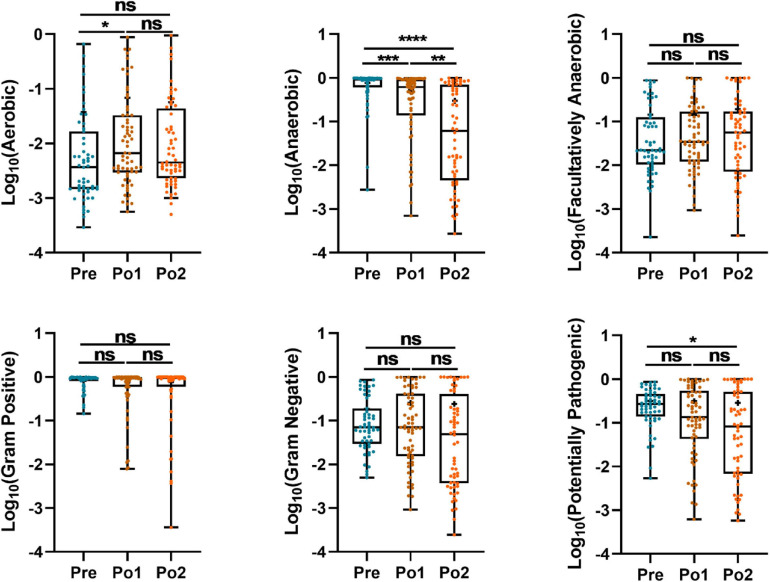
Perioperatively BugBase analysis based on 16S rRNA gene sequencing of cohorts. Outcome was grouped according to time (*x*-axis). Logarithm of relative abundance with 10 as base is presented on y-axis. Statistical assessment was carried out with Kruskal–Wallis test followed by *post hoc* test using Dunn’s multiple comparison test. *P* < 0.05 indicates statistical significance (*P < 0.05, **P < 0.01, ***P < 0.001, and ****P < 0.0001), and ns means *P* > 0.05. Results are expressed as median and quartile.

To further investigate the feature of the gut microbiome, BugBase analysis, based on 16S rRNA gene sequences for analyzing the phenotype of the endophytic bacterial community, was carried out to predict the proportions of aerobic, anaerobic, facultatively anaerobic Gram-positive, Gram-negative, and potentially pathogenic microorganisms (Figure 5). The relative abundance of anaerobes steadily decreased over time; however, the relative abundance of aerobes in Po1 was increased compared with Pre. The potential pathogenic microbiome in Po2 was lower than that in Pre. Hence, the postoperative fecal microbiome was characterized by decreased anaerobes and increased aerobes.

### Fecal Bacterial Taxa Were Influenced After Cardiovascular Surgery

**FIGURE 6:**
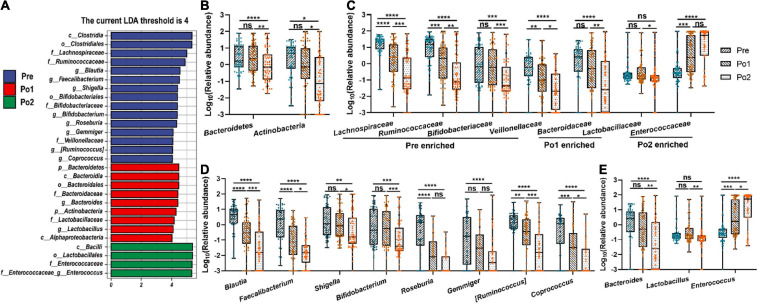
Microbial differential analysis and changes of specific bacterial taxa. Linear discriminant analysis coupled with effect size analysis **(A)** and comparison of representative taxonomic abundance in phylum **(B)**, family **(C)**, and genus level **(D,E)** were assessed across time series. LDA discriminant bar counts biomarkers with significant effects in different groups with an LDA score greater than 4. Statistical assessment was carried out with one-way analysis of variance or Kruskal–Wallis test for parametric or non-parametric data followed by *post hoc* test using Dunn’s multiple comparison test. *P* < 0.05 indicates statistical significance (*P < 0.05, **P < 0.01, ***P < 0.001, and ****P < 0.0001), and ns means *P* > 0.05. Results are expressed as median and quartile.

To further identify distinct bacterial taxa among the different perioperative periods, we performed linear discriminant analysis coupled with effect size analysis and identified two phyla, seven families, and 11 genus showing significant differences (Figure 6A). At the phyla level, *Bacteroidetes* and *Actinobacteria* were enriched in Po1 (Figures 6A,B). At the family level, *Lachnospiraceae*, *Ruminococcaceae*, *Bifidobacteriaceae*, and *Veillonellaceae* were enriched in Pre, and *Bacteroidaceae* and *Lactobacillaceae* were enriched in Po1, whereas *Enterococcaceae* was enriched in Po2 (Figures 6A,C). Furthermore, genus *Blautia*, *Faecalibacterium*, *Shigella*, *Bifidobacterium*, *Roseburia*, *Gemmiger*, [*Ruminococcus*], and *Coprococcus* were enriched in Pre (Figures 6A,D). *Bacteroides* and *Lactobacillus* were enriched in Po1, whereas *Enterococcus* was profoundly increased after surgery and enriched in Po2 (Figures 6A,E). Therefore, these results indicated that the gut microbiome of patients was drastically altered during perioperation; in particular, *Enterococcus* was significantly increased after surgery.

### Perioperative Change of Fecal Microbiome Was Associated With Systemic and Intestinal Inflammation

**FIGURE 7:**
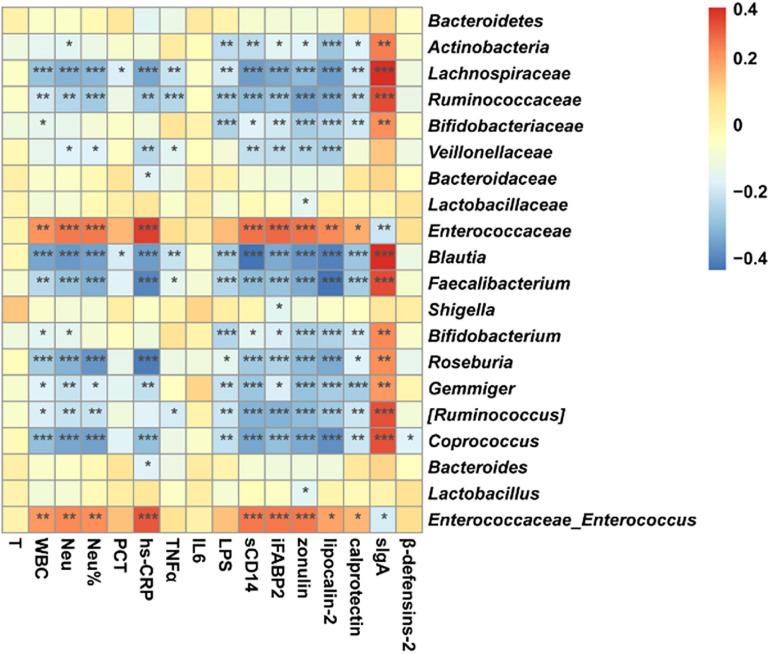
Correlation between microbiome and parameters of systematic infection, plasma pro-inflammatory cytokines, intestinal barrier, local intestinal inflammation, and gut immunity. Star means a significant correlation, whereas *P* < 0.05 indicates statistical significance (*P < 0.05, **P < 0.01, and ***P < 0.001). Orange represents negative correlation, and blue represents positive correlation.

Spearman correlation analysis was carried out to evaluate the potential relationship between the microbiome and the parameters of systematic infection, pro-inflammatory cytokines, gut barrier, and intestinal inflammation and immunity (Figure 7). *Enterococcaceae* and *Enterococcus*, which were Gram-positive, facultatively anaerobic, and opportunistic pathogens, were positively correlated with WBC, Neu%, hs-CRP, sCD14, iFABP, zonulin, lipocalin-2, and calprotectin, meanwhile negatively correlated with sIgA. In contrast, *Blautia*, *Faecalibacterium*, *Bifidobacterium*, *Roseburia*, *Gemmiger*, [*Ruminococcus*], and *Coprococcus*, which were strictly anaerobic or short-chain fatty acid-producing bacterium, were negatively correlated with indicators of systematic infection, gut barrier, and intestinal inflammation and immunity. Therefore, microbiome change was related to systematic infection and gut dysbiosis during perioperation time.

## Discussion

In this study, we disclosed that the fecal microbiome of patients after cardiovascular surgery was disordered compared with the preoperative stage, and accordingly, postoperative patients had increased systemic inflammatory response and imbalanced gut homeostatic. Importantly, we figured out that the decrease of anaerobes and expansion of *Enterococcus* were positively related to increased systemic inflammatory response and imbalanced gut homeostatic, suggesting that there is probably some relative among gut microbiome, intestinal inflammation, and systemic inflammatory response.

To our knowledge, this study firstly depicted the longitudinal change of gut microbiome in patients during the perioperative period of cardiovascular surgery. The decrease of alpha diversity and anaerobes and the increase of *Enterococcus* were the main characteristics of intestinal microbiological changes after surgery. As is well known, cardiovascular patients receiving general anesthesia, supported by cardiopulmonary bypass or not, would suffer surgical trauma and ischemic intestinal reperfusion injury, which had been confirmed as disturbing factors of intestinal microbe (Tian et al., 2016; Malan-Muller et al., 2018; Serbanescu et al., 2019). Besides, the application of antibiotics and the environment of a hospital would also disrupt the gut microbiome (Ruppe et al., 2018; Fassarella et al., 2020; Zheng et al., 2020). Likewise, critical status after cardiovascular surgery resulted in severely pathophysiologic changes and had been reported to drive drastic variation in the gut microbiome (Dickson, 2016). In the current study, imbalanced gut immunity of gastrointestinal, indicated by decreased sIgA and elevated β-defensins 2, was observed, which may also contribute to a dramatic change of fecal microbiome in patients (Lindner et al., 2015).

In this study, decreased alpha diversity of the microbiome, as the result of microbiome disorder after cardiovascular surgery, was associated with enhanced gut mucosal inflammation indicated by increased fecal calprotectin and lipocalin-2. These findings were consistent with other animal models and human studies (Lapthorne et al., 2013; Yeh et al., 2016). In addition, we found that the anaerobes, the major commensal bacteria, were decreased after cardiovascular surgery. *Blautia* had been observed as a beneficial anti-inflammatory in several clinical settings and was associated with improved outcomes (Jenq et al., 2015). *Faecalibacterium*, [*Ruminococcus*], and *Coprococcus* are considered butyrate-producing bacteria (Fu et al., 2019). *Bifidobacterium* is a major anaerobic probiotic in the human intestinal, which can exert health-promoting effects to stimulate mucin production, produce short-chain fatty acids and other antimicrobial substances, and protect against human pathogens (Hidalgo-Cantabrana et al., 2017). *Roseburia* is obligate Gram-positive anaerobic bacteria and a part of commensal bacteria producing short-chain fatty acids, especially butyrate. *Roseburia* can affect colonic motility, immunity maintenance, and anti-inflammatory properties (Tamanai-Shacoori et al., 2017). Therefore, the anaerobes and health-associated microbiota are not only important in maintaining gut barrier integrity, anti-inflammatory effects, and immune balance but also play a key role in maintaining a positive microbial environment in the gut (Bian et al., 2020; Gao et al., 2020; Jiang et al., 2020; Seo et al., 2020), and their depletion may be a potential inducement of elevated postoperative systemic and gut local inflammation.

Furthermore, *Enterococcus*, which belongs to facultative anaerobe, was markedly enriched and dominant in Po2. These pathogens in the hospital setting are primarily related to their survival capabilities in a hostile antimicrobial-rich environment (García-Solache and Rice, 2019). A study on mice showed that *Enterococcus faecalis* could produce metalloprotease gelatinase triggering intestinal inflammation, thereby impairing epithelial barrier integrity (Steck et al., 2011). Another research also demonstrated that specific *Enterococcus* could generate a microbiome-derived toxin and translocate to blood under the circumstance of the impaired gut barrier (Duan et al., 2019). Moreover, two major species of *Enterococcus* demonstrated intrinsic resistance to common antibiotics and were frequent causes of hospital-acquired infections, which would cause poor prognosis of inpatients (Gao et al., 2018; García-Solache and Rice, 2019). In this case, fecal *Enterococcus* may be served as an indicator for monitoring the disorder of intestinal microbiome in patients who underwent cardiovascular surgery. However, further study should be carried out to figure out which specific strain and mechanism were involved in the damage of the intestinal barrier.

The resulting inflammatory response is related to postoperative complications such as atrial fibrillation, respiratory failure, acute kidney injury, neurologic dysfunction, myocardial dysfunction, liver dysfunction, and even multiple organ failure (Rinder, 2006; Song et al., 2008; Kraft et al., 2015). These complications have been reported to increase morbidity and mortality rates of cardiovascular patients after surgery (Rinder, 2006; Kraft et al., 2015). Therefore, elucidating the possible mechanism of systemic inflammation after cardiovascular surgery is of great significance to improve the clinical outcome.

During the perioperative period, various factors, such as blood exposition to the non-endothelial surface, ischemia–reperfusion injury, anesthesia, blood transfusion, usage of extracorporeal membrane oxygenation and intra-aortic balloon pumping, etc., may trigger an elevated inflammatory response (Kraft et al., 2015), which will cause dysregulation of inflammatory mediators and activation of leukocytes (Vallely et al., 2001; Melley et al., 2005; Nicholson and Hall, 2011). In this situation, pro-inflammatory cytokines cause immune activation and disturbance in the intestinal barrier (Al-Sadi et al., 2013). Furthermore, microbiota composition and diversity are important in maintaining gut barrier integrity, anti-inflammatory effects, and immune balance, and gut microbiota disorder will deteriorate the impaired intestinal barrier and accelerate antigenic penetration into the underlying intestinal tissue. Finally, this would allow a portal influx of pathogen-associated molecular patterns to blood circulation, triggering a pro-inflammatory cascade and causing increased pro-inflammatory cytokines in plasma such as IL-6 and TNF-α (Albillos et al., 2020). Moreover, secretion of IL-6 and TNF-α would amplify leukocyte activation, leading to the elevation of WBC and neutrophil count, and also stimulates the liver to produce acute inflammatory phase proteins such as CRP (Fujishima and Aikawa, 1995). The pro-inflammatory cytokines produced during inflammatory response cause disruption of the intestinal barrier resulting in a further increase in intestinal permeability (Shen and Turner, 2006), creating a vicious circle among microbiota disorder, gut dysbiosis, and systemic inflammatory response. This is in line with our results, indicating imbalanced gut homeostasis resulting from gut microbiome dysbiosis after cardiovascular surgery that would lead to the elevated plasma endotoxin, which was a potential mechanism of increased systemic inflammatory response.

There are still some limitations in this study. Firstly, the microbiome analysis based on 16S rRNA gene sequencing was insufficient to resolve accurate species, strains, and microbiome functions. Furthermore, viruses and fungi were not assessed, which may also lead to adverse clinical events. We did not set the control, health volunteers, or patients admission to ICU without surgery. Another limitation is that it was impossible to distinguish the individual factors that lead to changes in intestinal microflora due to the ubiquitous use of antibiotics. We did not take into account all the potential factors influencing the gut microbiome, e.g., glucocorticoids and proton pump inhibitors. Despite the limitations, our research is favorable in understanding perioperatively dynamic changes of gut microbiome who undergo cardiovascular surgery. Based on our finding, *Enterococcus* is probably some relative to systemic and intestinal inflammation (Steck et al., 2011; Clokie, 2019; Duan et al., 2019), and this could be potential causality and intervention target, whereas the depletion of heath-associated taxa could be potentially useful probiotics to improve intestinal microenvironment. Besides, some other intervention manners that could regulate resident flora and enhance epithelial barrier function and regulate intestinal and systemic immunity would also work, such as probiotics, prebiotic fecal microbiota transplant, and so on (Staley et al., 2017; Salminen et al., 2021; Zhang et al., 2021). Therefore, our research proposes a promising perspective of potential diagnostic and therapeutic options for perioperative management of cardiovascular surgery patients.

## Conclusion

In conclusion, our study shows that the patients with cardiovascular disease underwent gut microbiome disorder after surgery, accompanied by a damaged gut barrier, imbalanced gut homeostatic, and increased systemic inflammatory response. Correlation analysis shows microbiome dysbiosis was positively related to increased systemic inflammation and intestinal dyshomeostasis, especially the expansion of *Enterococcus*. We conclude that microbiome dysbiosis contributes to increased intestinal inflammation and subsequently elevated plasma endotoxin and systemic inflammatory response after cardiovascular surgery. Further research should be carried out to elucidate the exact mechanisms perioperatively.

## Data Availability Statement

The datasets presented in this study can be found in online repositories. The names of the repository/repositories and accession number(s) can be found below: SRA database, accession PRJNA718417.

## Ethics Statement

The studies involving human participants were reviewed and approved by the Ethics Committee of Tongji Hospital, Tongji Medical College, Huazhong University of Science and Technology (Wuhan, Hubei, China). The participants and/or their families provided their written informed consent to participate in this study.

## Author Contributions

XX, XW, HW, and JP designed the experiments. XX, SY, and ZY collected the data. XX collected the samples and wrote the manuscript. XX and JN conducted the laboratory analyses. JP, HJ, XW, HW, and CW reviewed and edited the manuscript. All authors read, commented, and approved the final version of the manuscript.

## Conflict of Interest

The authors declare that the research was conducted in the absence of any commercial or financial relationships that could be construed as a potential conflict of interest.

## Publisher’s Note

All claims expressed in this article are solely those of the authors and do not necessarily represent those of their affiliated organizations, or those of the publisher, the editors and the reviewers. Any product that may be evaluated in this article, or claim that may be made by its manufacturer, is not guaranteed or endorsed by the publisher.
